# Mr. Spencer on Cynanche Trachealis

**Published:** 1812-01

**Authors:** Samuel Spencer

**Affiliations:** Duffield, near Derby


*To the Editors of the Medical and Physical Journal.*

GENTLEMEN,

DR. BEAVER merits the thanks of the profession for his candid statement of the case of Cynanche Trachealis, in an adult subject, which lately happened in his practice; if an inspection of the parts after death had been allowed, it would have been curious to have ascertained whether there was the same disposition to throw out coagulable lymph as happens in children under a similar disease of the same organs. Cynanche Trachealis, in the adult subject, is undoubtedly undoubtedly a disease of rare occurrence; so much so indeed as to induce Dr. Beaver to say, "notwithstanding my most minute research into medical histories, I have only met with one solitary case, and that in the person of the celebrated General Washington." I had an idea on reading Dr. Beaver's paper, that Boerhaave in his aphorisms, had distinctly described the same disease, and on referring to that admirable little work, I found it to be the case. The symptoms which he enumerates, exactly correspond with those which occurred in Dr. Beaver's case. The half-reclined posture of the body, to assist in the almost stifled respiration, the aversion to speaking, the shrill tone of voice, the anxious countenance, the difficult deglutition, without any external signs except those of fever, are all distinctly marked by him as symptoms of this disease. And, morever, his learned commentator recites a case from Tulpius, in which the most timely, rational, and decided, practice gave no relief; and, he says too, that "a like case came under my own observation, in a man of fifty years old, ill of a fever, which, though not intense, was attended with an obstruction of the swallowing, and a most acute squeaking voice." And, he further tells us, that the celebrated author of the Aphorisms above alluded to, "knew a man seized with the like disorder in the midst of a feast, and, while the company were thinking that the unhappy sufferer feigned such a sharp voice for the sake of mirth, he was suffocated before any remedy could be applied."

If you think that a knowledge of these facts will be satisfactory to Dr. Beaver, you will give them, I hope, a place in your valuable miscellany. I am, &c.

SAMUEL SPENCER.

*Duffield, near Derby, Nov.* 10, 1811.

